# The socio-psychological factors affecting the voting behaviour of the postgraduate politics students: a Q-methodology study

**DOI:** 10.3389/fpsyg.2023.1218104

**Published:** 2023-12-04

**Authors:** Turan Şener, Yaşam Balku, Yavuz Selim Alkan, Serkan Doru, Kadriye Okudan Dernek, Samet Zenginoğlu

**Affiliations:** ^1^Department of Political Science and Public Administration, Akdeniz University, Antalya, Türkiye; ^2^Department of Economics, Adıyaman University, Adıyaman, Türkiye

**Keywords:** political psychology, voting behaviour, socio-psychological theory, rational theory, Q-methodology

## Abstract

This study aims to answer the question “Are voting behaviours of postgraduate students, a voter group who are politically educated and well-informed about voting behaviours, affected by socio-psychological factors?.” In particular, if so, it also aims to reveal which socio-psychological factors affect their voting behaviours. The Q-methodology is utilised in this study. The main reason for this methodological preference is that the Q-methodology is a good tool for systematically identifying and examining a particular group’s subjective views that are held around the factors shaping and affecting their voting behaviours by providing factor loadings. Factor loadings, or the cluster of participants, allow us better to illustrate each participant’s association with each of the identified socio-psychological or otherwise factors, similar or different orders of ranking by the participants, to detect individual differences, and, therefore, to indicate (1) whether the socio-psychological factors affect the voting behaviours of the participants, and (2) if so, which socio-psychological factor(s) affects most. This also helps us to conclude that the participants who are mostly associated with one or more factors have similar voting behaviours corresponding to or in opposition to the assumptions of the socio-psychological approach. The participants of the research are 57 postgraduate students studying Political Science and Public Administration at Akdeniz University. The results of the research indicate that most of the participants cluster around two separate factors: while the participants gathered under Factor 1 take their political decisions more rationally and are ready to vote for alternative candidates and political parties in different elections, those who load under Factor 2 are affected by some socio-psychological factors: loyalty to her family’s (the family factor) and inner circles’ political preferences (the inner circle factor), and a long-term commitment and an emotional attachment to a political party and/or the candidate (the time factor). The voting behaviours of the participants gathered under no factors are, however, affected by relatively mixed factors. In addition, it is also revealed that the titles that most differentiate the preferences of the participants cluster around both Factors 1 and 2 are family, education, and rationality.

## 1 Introduction

Individuals make choices among various alternatives throughout their life spans. These preferences may be a direct choice of a particular material element, benefit, or interest, or a preference for an intellectual, metaphysical, moral, or ethical issue. Individuals can make these decisions not only individually but also collectively through the determination and aggregation of individual votes and preferences and the determination of the majority vote. Accordingly, decision-making can be manifested in all areas of private or public life. No matter in which field they occur, all these decision-making and voting processes are influenced by a wide range of factors. Individuals make their decisions and cast their votes under the influence of a great number of personal, group, or society-related, psychological, political, sociological, or cultural factors. Voter behaviour approaches in different areas accordingly aim at revealing those divergent factors, developing their claims, arguments, and assumptions referring to different, mostly conflicting, theoretical underpinnings, and applying numerous research techniques and methods.

Bearing in mind the fact that identifying all factors influencing voting behaviour in all different areas is an arduous task, the following example can be given: Focusing on corporate governance and shareholder meetings, Dressler and Mugerman (2023) analyse the impact of voting power on the voting behaviour of shareholders at general meetings. They offer the following factors that shape voter behaviour with reference to a comprehensive literature review: outcome preferences, peer effects, self-interest, normative considerations, and voting power (2022, p. 1089). They also review models aiming to express the motivations behind voting behaviours of individuals: instrumental voting, based on the assumption that “a voter’s behaviour is rational and designed to maximise value,” and expressive voting, which claims that motivation for voting is not only about the ultimate outcome but also about “the significance of the very act of voting,” i.e., one may vote with the aim of expressing her own identity or moral view (2022, p. 1092; see also Ferejohn and Fiorina, 1974; Shayo, 2009; Shayo and Harel, 2012; Yin et al., 2021).

Dressler and Mugerman’s (2023) aforementioned categorisation raises a crucial issue: The factors that motivate or influence voting are not only rational but may also be irrational, intangible, or emotional. This issue is particularly controversial in political science, where a vast scholarly literature has been devoted to the examination of rational and/or opposite factors affecting individual or collective voting behaviour, especially after the rise of postpositivist and postmodern theoretical and normative perspectives in political sciences (see Key, 1966; Kramer, 1971; Inglehart and Sidjanski, 1976; Books and Prysby, 1988; Inglehart and Norris, 2000; Duch and Stevenson, 2008; Bartels, 2010; Sharlamanov and Jovanoski, 2014; Mahsud and Amin, 2020). Among these theoretical and empirical efforts, for instance, Kulachai et al. (2023) provide a meticulous and comprehensive review of factors affecting the voting decision. Even though they determine the essential factors, focusing particularly on the American populace, their factors can be generalised owing to their attempt to compile not only interrelated but also conflictual factors and the fact that they synthesise various theoretical perspectives and empirical studies. Kulachai et al. (2023) categorise factors affecting voting behaviour into three main categories, each of which contains sub-factors: individual-level factors (income, education, gender, age, political ideology, personality traits, emotional intelligence (EI), climate change concerns, and healthcare experiences); socio-cultural factors (social identity, ethnicity and race, religion, media influence, and social networks); and political factors (party identification, candidate characteristics, policy positions, campaign strategies, and economic conditions). They conclude that, despite the abundance of divergent factors, every single factor is intertwined and interacts with others, and the relative significance of these factors varies depending on the individual and the context.

Inspired by Kulachai et al.’s (2023) aforementioned factors, which are open to contextual differences, we prefer to examine the voter behaviour approaches, dividing them into the following three main types: the socio-psychological approach (Campbell et al., 1960), the rational approach (Downs, 1957), and the sociological approach (Lazarsfeld et al., 1968). The socio-psychological approach (Campbell et al., 1960), in particular, purports to explicate factors affecting voting behaviours assuming that several socio-psychological factors affect the voting behaviour of the electorate, including the family, candidate, ways of persuasion, and political campaigns. This approach, in other words, essentially claims that the political attitudes, ideologies, and party preferences of individuals are extremely influenced by their childhood experiences, political preferences, and the tendencies of both their families and inner circles. Factors highlighted by the socio-psychological approach, along with, but not limited to, other factors determined by the aforementioned two approaches, are of great interest to researchers, political psychology enthusiasts, and policymakers. The question of factors influencing voting behaviour is a substantial one, with far-reaching implications for a variety of stakeholders.

In this sense, this study is motivated by a similar question and aims to reveal the socio-psychological factors affecting the voting behaviours of postgraduate political science students. These students might potentially be expected to make political decisions or select among divergent policy issues, candidates, or political parties, especially under rational factors since they are both having official and scientific political science education and academically well-informed about the theoretical and practical aspects of voting behaviours, rational approach, and its alternative perspective, the socio-psychological approach. This study aims to explore whether the opposite is possible. To that end, this study aims to reveal the subjective viewpoints of postgraduate students on the socio-psychological factors affecting their voting behaviour. Detecting the socio-psychological factors that have consensus or that the participants agree on enables us to conclude that the participants loading under similar factors have similar voting behaviours and are affected by similar socio-psychological factors while making their political decisions.

For this purpose, the Q-methodology is particularly used in this study to identify similar or different overall attitudes of the participants concerning the socio-psychological factors affecting voting behaviour. The Q-methodology aims to detect agreements/consensus, and disagreements between the individual and group of participants on a particular topic (Brown, 1980; Lecouteur and Delfabbro, 2001; Gao and Soranzo, 2020) by examining their similar or different self-expressed subjective points of view through a holistic approach (Brown, 1993; Watts and Stenner, 2005a). In other words, the Q-methodology aims to discover common viewpoints obtained from subjectively delivered viewpoints by combining both qualitative and quantitative methods (Watts and Stenner, 2012). Since it enables the participants to compare all the statements systematically, the outcome of the Q-sorting is “based on a holistic thinking process rather than isolated ratings” (Gao and Soranzo, citing Watts and Stenner, 2012). This holistic approach helps quantify “patterned subjectivities” (Shemmings, 2006, p. 147). Even though participants can subjectively rank the factors by significance in a great number of different ways, “the resultant factors may [still] represent common viewpoints” (Karasu and Peker, 2019, p. 41). Besides, as Gao and Soranzo (2020, p. 2) aptly compile, when compared to the other methods, i.e., Likert attitude questionnaires, the procedure of Q-sorting is “less time-consuming, more engaging for participants and more natural,” allows the participants to “accurately differentiate the subtle differences in their judgments,” and guarantees “a more coherent and accurate analysis of the decision process” by minimising “the order effect.” In addition, as ten Klooster et al. (2008, p. 512) note, even though it is possible to utilise many other segmentation techniques, i.e., cluster analysis and discriminant analysis, to examine the survey data, they are relatively “more complex and their interpretation is less straightforward.”

Given the aforementioned aim of this study, the Q-methodology allows us to reveal the subjective viewpoints of the postgraduate students on socio-psychological factors and, therefore, determine whether and which socio-psychological factors affect their voting behaviour most. The sample of the study consists of 57 postgraduate students who are currently doing either Master’s or Ph.D. studies in Political Science and Public Administration at Akdeniz University, Institute of Social Sciences. Since the research is principally organised within the framework of the theoretical essentials and assumptions of the socio-psychological approach, the Q statements and titles are formed and presented to the participants concerning the socio-psychological approach. The analysis of the data is carried out using the “PQMETHOD 2.35,” specific computer software for the Q methodology.

As to this study’s contribution to the existing literature, to the best of our knowledge, the Q methodology has not yet been widely used in political sciences, public administration, or political psychology, except in the works of Brown et al. (1999, p. 605), Durning (1999), Webler et al. (2001), and Lehtonen and Aalto (2016). Given the aforementioned advantages of using the Q methodology, this study might provide a ground for further studies aiming to detect similar or different factors, tendencies, and perceptions of the voter groups. The findings of this study might also have potentially broader implications: by utilising an alternative method and revealing that a “modernised” and “rational” group of people can still make political decisions under emotional factors, this study might allow for a deeper analysis of voter behaviours in the political sciences. Its findings might potentially be used to challenge modern/positivist assumptions regarding voting behaviours and patterns. In addition, given that today’s late (post)modern world is defined in terms of criticism of representative democracy (for a review, see Alkan, 2018) diversity, multiculturalism, and respect for differences, it is vital to understand the preferences, interests, and choices of (post) modern individuals, groups, or communities as well as the emotional or less rational factors affecting their formation processes in order to formulate more inclusive and democratic decision-making processes. It is another and comprehensive question that the results of this study can be interrelated to is to what extent and under what conditions emotions, passions, and arguments derived from religious, ethical, or moral values can enter the public political debate (see Habermas, 1995; Mouffe, 2002; Rawls, 2005; Alkan, 2015).

In this context, this study is structured in three parts. The first part starts with an explanation of the characteristics of the socio-psychological voter approach. In this section, the focus is given to the main theoretical assumptions of the socio-psychological approach. The rational and sociological theories are particularly examined in this section only when necessary. Thereafter, it proceeds with providing the details of the research method adopted. The last part is allocated to the findings and the concluding remarks.

## 2 Materials and methods

### 2.1 Theoretical background: voter behaviour and the socio-psychological approach

The socio-psychological approach is first established and then developed as a result of a great deal of empirical research conducted by the National Center for Election Studies, which was established at the University of Michigan in the 1950s. An influential comprehensive book, The American Elector, provides an examination of data collected and analysed within the aforementioned research efforts by Campbell, Stokes, Converse, and Miller. This study is principally an analysis of data obtained from interviews made with individuals who expressed their views and preferences about the presidential elections held in 1948, 1952, and 1956. Campbell et al. determined that some individual socio-psychological factors shape the preferences of voters in these three elections (Campbell et al., 1960, p. 10; Pomper, 1978, p. 618; Akgün, 2002, p. 78; Kavas, 2017, p. 79). For this crucial reason, the socio-psychological approach focuses especially on individuals rather than groups in terms of factors affecting voter behaviours. The approach essentially propounds that the political attitudes, ideological, and party preferences of individuals are highly influenced by their childhood experiences, political preferences, and tendencies of both their families and inner circles (see Gökçe et al., 2002, p. 8; Sears and Brown, 2013, p. 3).

Before the emergence of the Michigan approach, researchers, who examined the factors that affect the politicisation process of individuals from an early age, already focused on and pointed out the effects of the inner circles, such as family and friends, on the individuals’ political preferences and behaviours. The Michigan approach, however, makes the distinction between the party identity and the party identity coming from the past concerning the family factor (Ventura, 2001, p. 667). In other words, the psychological process that a person experiences throughout her life includes the way she interprets political events and her political attitudes (Milburn, 1998, p. 274). According to the socio-psychological approach, the individual begins to sympathise with a particular political party in the early childhood period. According to Akgün (2002, p. 26), the sympathy of the individual at an early age with a political party causes her to be psychologically attached to that party in the future. This, therefore, makes voting behaviour a personal attribute that can be transferred from generation to generation.

According to the socio-psychological approach, personal childhood experiences are the main factors affecting voting behaviour. Voters are affected by their early childhood lives and experiences while voting for a political party (Evans, 2004, pp. 23–24). The main factors that establish the bond between an individual and her preferred political party are emotional states such as loyalty, love, and support (Pomper, 1992, p. 35). For this reason, according to Campbell et al. (1960, p. 58), the bond of love to a political party deepens over time and leads to the development of partisanship. The partisan political attitude enables the voters to vote for the party that they find psychologically suitable for them. This attitude, which is also called identification with a political party, is, therefore, a reflection of the politicisation process that individuals acquire from an early age. The individual, who is attached to the political view of her family with strong psychological bonds, cannot easily break this bond in adulthood. The individual always continues to be under the strong influence of her own social environment (Campbell et al., 1960, p. 147).

The basic assumptions of the socio-psychological approach can therefore be summarised as follows:

- For most of the voters, alongside the party they currently choose to vote for, there may also be a different party with which they previously felt emotional attachment.
- This pre-existing emotional attachment is formed by family-based socialisation.
- Most voters stay loyal to the same political party their entire lives.
- The minority group whose party affiliation has changed over time is the group that has either very weak family ties or does not ever have these ties. This group deviates from the majority because their families’ original affiliations are different from the source groups.
- It is possible for voters who are more loyal to a party to vote independently for that party in the elections.
- Voters who are more loyal to a party are also likely to participate in the elections.
- Temporary inconsistency between voting for a party and identifying with a party may occur due to the attractiveness of candidates and topics. However, this situation continues for one or two elections only, and in the following periods, the person generally votes by party affiliation.
- It is possible for voters who are more loyal to a party to perceive and evaluate other parties that are suitable for their partisan affiliation (Kalender, 2014, p. 46; see Budge and Farlie, 1997, pp. 42–43; Fiorina, 1997, pp. 391–414).

According to the socio-political approach, when evaluating the election campaigns, voters are inclined to vote for the party with which they have been affiliated since childhood and which is most suitable for their psychological disposition. These voters can choose either the party they preferred in the past elections or a new party that has some affinities with the party they are already loyal to (Smith, 2001, p. 294). It can be deduced from this that even if the voters support different parties for a short time, they may start to prefer the party they used to vote for again (Harrop and Miller, 1987, p. 134). In addition, when a previously attached party has no chance to make a change in government formation, it would still be preferred, or an alternative party would take its place emotionally as a substitute according to the socio-psychological approach. In other words, despite some short-term changes in one’s political preferences, there is generally only one political party that the voters support constantly.

While analysing the socio-psychological bases of the 1952 elections in their monumental study titled The Voter Decides, Campbell et al. (1954) observe that the most significant factor affecting voting behaviour is the perceptions of the people rather than their demographic status and social position. The voter prefers one candidate or political party to another according to her own perceptions and attitudes (Asher, 1980, p. 36). Under the psychological effect of sympathy coming from her past experiences, voters may cast their votes feeling an emotional affinity towards the party’s candidates or even the party’s logo (Akgün, 2002, p. 26). The evaluation process of the voter is, therefore, more emotionally based than rational.

The fact that voters evaluate the party or candidate rationally, not emotionally, is the main factor that differentiates rational choice theory from socio-psychological theory (Harrop and Miller, 1987, p. 146). Downs (1957), who is one of the pioneers of the rational approach, argues in his classic called An Economic Theory of Democracy that voters may not vote for the ruling party or candidate in power if they do not have a good chance of winning (1957, p. 50). In a similar vein, she may choose not to vote if she finds the implemented policies unsuccessful or the promises given are not fulfilled.

One of the principal assumptions of the rational approach is that voters follow a rational and strategic evaluation process while making their decisions. According to the rational approach, the voter strategically evaluates the promises and winning potential of the candidate and weighs what the candidate has said and done before and after the election. The socio-psychological approach, on the contrary, claims that voters generally prioritise their emotional closeness to the candidate or political party over the rational evaluation process. The voters, in other words, make their decision in the elections by putting the candidate’s voting potential in second place.

The education/knowledge factor can also be included in the rational evaluation process. It is contended that as the level of knowledge of the voter increases, voting behaviour turns into strategic behaviour, in other words, she moves away from acting emotionally and prefers the party that is most suitable for her own benefit (Gülmen, 1979, p. 27). In a similar vein, as the voters become much more rationalised and, at the same time, have a significant amount of knowledge about the economy, they prefer to vote according to their evaluations of the course of the economy to maximise their own interests (Holbrook and Garand, 1996, p. 367). The researcher should know the level of knowledge of the voter in order to be able to effectively discuss whether an individual is rational (Gülmen, 1979, p. 27).

The socio-psychological approach assumes that the voter is less rational but more emotional than supposed and that she is much more affected by the reference groups. To put it differently, according to Shachar (2003, p. 252), voting behaviour is mostly hereditary. The voter’s decision about which candidate or political party to support has already taken place in her mind due to a significant reference group, namely her family’s long-standing influence on her political decisions. On the other hand, the groups that directly affect this emotional evaluation process are not only the families of the voters, but they are also the religious or social groups to which the voter belongs. In addition, voters may also be under the influence of inner circle relations (Bartle, 1998, p. 511). It should be emphasised that the sociological approach differs from the socio-psychological approach at this crucial point: the former only examines why the individual acts in line with the group’s political preferences. The sociological approach, developed by Lazarsfeld et al. (1968, pp. 137–140), for instance, claims that the individual reflects the voting behaviour of the group she is affiliated with. Individuals may therefore have similar political preferences and tendencies with their family, friend group, or community. It should be noted that in addition to exhibiting similar voting behaviours to the group’s political preferences, individuals may also tend to vote for the candidate or political party preferred by the majority.

The influence of the reference group on the individual’s voting behaviour may lead her psychologically to vote for a candidate that reflects the characteristics of the group. This is mainly because, as the socio-psychological approach proclaims, the voters evaluate the attitude and image of the candidate emotionally. Voters may find some candidates much closer to them who imply certain traits of their group, such as religion, language, race, or ethnicity. Voters believe that candidates who have similar personality traits to theirs can only genuinely represent them. This tendency may also be affected by the way the candidate takes part in the social environment and the candidate’s interests (Manza and Brooks, 1997, pp. 72–74).

The image of the candidate can also affect the psychological decision-making process of the voter. Newman (1994, p. 72), for instance, claims that the likelihood that a candidate with a positive attitude might get more votes than any other is significantly high. Other qualities that positively affect the decision of the voters are related to the fascination, honesty, and personality of the candidate (Shachar, 2003, p. 252).

Other emotional factors, such as beliefs, perceptions, needs, and motives, can also be included in the category of psychological factors. Among these psychological factors affecting the voters’ political preferences, one of the most prominent is persuasion. Through political and election campaigns, the perceptions of the voters can be influenced, and the voters can be persuaded to vote (Şener, 2018, p. 117; Aydin and Sener, 2018). Political campaigns can either reinforce or change the voter’s opinion or mobilise the voter (Lazarsfeld et al., 1968, p. 103). As stated by Campbell et al. (1960, p. 66), even if they have short-term effects, persuasion campaigns can influence the decisions and preferences of the voters. Voters can be convinced by taking into consideration the personality traits of the candidates, their capacity to represent the group’s interests, or by comparing the political promises and public policy projections. On the other hand, even though persuasion is categorised as a socio-psychological factor in this study by particularly embracing the approach of Campbell et al. (1960), persuasion can also be accepted as a rational factor: facts, data, and other relevant information acquired by adopting rational and scientific methods can be used to influence the preferences, behaviours, and decisions of an individual or a group of people. Both categorisations are correct. In this study, however, persuasion is regarded as a socio-psychological factor due to its ethical and emotional dimensions and the fact that political actors can appeal to both rational and irrational beliefs of voters by using both rational and irrational ethical and moral tools. The use of consent and persuasion as both democratic and authoritarian/totalitarian tools is always possible.

### 2.2 Research method

Given the aforementioned theoretical framework, this study aims to determine the factors affecting the voting behaviours of a particular group of postgraduate students who are academically informed about their voting behaviours, within the framework of socio-psychological theory. In addressing this, the Q methodology is employed. Q-methodology is a systematic research method that makes it possible to analyse the beliefs, attitudes, approaches, and perceptions of participants using some measurement tools (Brown, 1993, 1996; Watts and Stenner, 2005b; Thompson et al., 2006; Demir and Kul, 2011; McKeown and Thomas, 2013; Yıldırım, 2017, p. 237).

The Q methodology was first devised by Stephenson (1935a,b, 1953), has been further developed (see Brown, 1993, p. 92; McKeown and Thomas, 2013), and has been intensely used by researchers in the fields of sociology and psychology since the 1940s (Nyumba et al., 2018, p. 21, e.g., Hedges, 2014; Gao and Soranzo, 2020). Its application and data analysis capabilities are also being extended and developed (e.g., Gao and Soranzo, 2020). Q methodology, as mentioned before, has also been applied in the political sciences.

As Coogan and Herrington (2011, p. 24) note, the development of the method arose from Stephenson’s ambition to apply a scientific framework to the elusive nature of subjectivity. For this purpose, he devises a methodology that enables individuals to reflect their point of view in order to retain it stable for investigation and comparison. It is an inverted technique of conventional factor analysis (ten Klooster et al., 2008, p. 512). The core of this method is that the person, not the test, is variable. In other words, it does not look for patterns among people but looks at the data in terms of an individual’s overall response patterns, or self-references.

As an alternative derivation of SPSS, the Q-analysis (Brown, 1993, p. 92) is a factor analysis that allows public administration and many other fields in social science to objectively determine the divergent psychological perspectives (Brown et al., 1999, p. 599; Webler et al., 2009, p. 6). Since it obtains data by discovery, the method is an embedded theory (Watts and Stenner, 2005a, p. 88). In this respect, it is a hybrid method since it includes both qualitative and quantitative methods of analysis (Stenner and Stainton Rogers, 2004, pp. 167–168; Ramlo and Newman, 2011, p. 173). One of the significant purposes of the method is to illustrate the subjectivity of individuals (Stergiou and Airey, 2011, p. 313) and to identify, if any, the subjective common, dominant (Van Exel and De Graaf, 2005, p. 1), or different (Zabala, 2014, p. 163) opinions within the group. In other words, the Q-methodology can reflect the perspectives of individuals on a certain topic by identifying the clusters of participants who generate similar results. By adopting a “bottom-up approach” to discover the individual differences in a group of participants (Gao and Soranzo, 2020, p. 3), Q studies, as Coogan and Herrington (2011, p. 24) point out, investigate relationships between individuals or entire facets of people’s thoughts and beliefs. Instead of testing the participants or enforcing pregiven meanings, participants are rather asked to determine what is meaningful to them. Thus, as Danielson (2009, p. 221-222) puts forward, a well-selected sample group might illustrate the leading perspective of the group.

The main stages of conducting a Q study can be briefly provided as follows, based particularly on Coogan and Herrington’s (2011, pp. 24–27) framework. As explained in more detail in the data collection, data analysis, and findings part of this study below, the first step is to create an appropriate set of statements derived from the existing concourse around the issue under investigation. The concourse refers to the compilation of relevant ideas, beliefs, and opinions regarding the research topic based on divergent sources, i.e., content analysis (ten Klooster et al., 2008, p. 512). The statements should be representative of the topic, reflect divergent vantages, include different sub-issues, and come from various sources, such as through interviews (see also Watts and Stenner, 2005a, 2012). The statements are then categorised, sub-categorised, duplicated, and formed as idea cards – all of the statements after this stage are called Q-sets. Running a pilot study is mostly deemed necessary. After generating the statements, the participants are asked to sort the idea cards on a Q-grid, including the “terms of references,” such as “most agreed/disagreed or most like me/least like me,” until no column is left empty (see Dennis, 1986). This is one of the crucial stages of the Q study because the ordering of the statements itself indicates the subjectivity of the participants. After this stage, the completed Q-grid is called Q-sort, which represents the subjective perspective of the participant on the investigated topic. A correlation matrix is then performed using the Q-sorts of all participants to show the degree of correspondence between the participants, and then it is subjected to a by-person factor analysis to investigate attitudinal groupings – to reveal one representative Q-sort per group, confounding and non-significant respondents (ten Klooster et al., 2008, p. 512). Finally, similarities and differences among the factors are interpreted and expressed (ten Klooster et al., 2008, p. 512). Data analysis is made by optionally using a software programme, i.e., PQMethod 2.11/2.35 (see Van Exel and De Graaf, 2005; Watts and Stenner, 2005a; Schmolck, 2014). Unlike other conventional attitude research approaches, the Q-sort method includes an inversion of the data matrix “so that the respondents are the variables, and the items [so to speak, statements] are the cases.” In other words, “respondents are correlated, instead of items” (ten Klooster et al., 2008, p. 512).

The factor analysis reveals similar orders of ranking by the participants adopting a holistic approach. In other words, participants with similar rankings of statements, or, in other words, with common similar subjective views on the topic, load particularly on the same factor. As Karasu and Peker (2019, p. 47) summarise, factors are interpreted by correlating a factor with other factors, taking the participant’s comments and demographic information into consideration, determining the distinguishing statements for every single factor, and/or the most agreed/disagreed statements and the Z-values of statements.

Accordingly, the Q-methodology is one of the best methods to identify the subjective views of participants on a particular issue. It “combines strengths of both qualitative and quantitative methods” (Brown, 1996, p. 561, see Dennis, 1988; Valenta and Wigger, 1997; Amin, 2000). It, in a word, quantifies “patterned subjectivities” (Shemmings, 2006, p. 147). As Coogan and Herrington (2011, p. 27) aptly claim, “[n]o other methods capture the essence of what the participants feel about a topic from collective voices, while at the same time identifying subtle differences between some of these voices.” This makes the Q-methodology an appropriate tool that should be utilised by political scientists who not only aim to identify the shared common political views and tendencies in a group but also detect and examine alternative political views and behaviours. The Q-methodology helps political scientists reveal whether a particular group has similar (or different) patterns of voting behaviour. Given the aim of this study and that it is less used concerning the topic and research question of this study, the Q-methodology is a good tool for systematically identifying and examining the postgraduate students’ subjective views that are held around the socio-psychological factors shaping and affecting their views, perspectives, and behaviours by providing factor loadings. Factor loadings allow us better to illustrate each participant’s association with each of the factors, similar orders of ranking by the participants, and, therefore, to reveal the statements that the participants have consensus on and to indicate which factor(s) affects most their views, perspectives, and behaviours.

In this context, the main research questions of this study that are formulated are as follows:

- To what extent are the socio-psychological assumptions in the socio-psychological theory of voter behaviour effective on the perceptions of the participants?
- Does a certain group of voters, who have sufficient knowledge about voter behaviour, tend to vote rationally or irrationally?
- Are there any certain socio-psychological factors affecting voter behaviour upon which the participants in the sample group agree and find most relevant? If so, what are these factors?
- Are there any certain socio-psychological factors affecting the voter behaviour upon which the participants in the sample group disagree and find most irrelevant? If so, what are these factors?

### 2.3 The sample

The Q-methodology enables researchers to conduct an empirical study with a small sample of participants (Dennis, 1988). This is mainly because samples in Q-sort studies are selected from a universe of perspectives, not from a population of people (ten Klooster et al., 2008, p. 513, citing Anderson et al., 1997). Thus, the number of participants in a Q-study could range from 40 to 60 (Brown, 1986; Dennis, 1988; Stainton Rogers, 1995; Karasu and Peker, 2019, p. 41). Thus, the sample of this research is 57 postgraduate students of the Department of Political Science and Public Administration of the Institute of Social Sciences at Akdeniz University, which is a state university located in Antalya, Türkiye.

Participation in the research is voluntary. The main reason for selecting the postgraduate students of the Department of Political Science and Public Administration is to determine the attitudes and perceptions of the voter group, which has completed an undergraduate degree-level course in political science and is undertaking further study at a more advanced level and has knowledge about the scholarly literature on voter behaviours. Accordingly, the participants in the study group are expected to meet the criteria “to have academic knowledge about voter behaviours” and “to be at the postgraduate level.” A total of 57 participants, 27 females and 30 males, who meet the criteria stated above, are included in the study group (Table 1).

**Table 1:** The socio-demographic profile of the participants.

Variable		Number	Percentage
Age	18–24	5	% 8,7
	25–30	33	% 57,8
	31–40	19	% 33,3
Sex	Male	27	% 47,3
	Female	30	% 52,6
Education	Master	36	% 63,1
	PhD	21	% 36,8
	Total	57	

### 2.4 Data collection

The Q-methodology consists of an index called Q-Sort and a certain number of positive and negative Q statements placed under pre-determined titles. The Q set (or Q sample) generally includes 40 to 50 statements, but as Van Exel and De Graaf (2005, p. 5) note, fewer or more statements can also be possible. In practice, even though most Q-Samples consist of 33 items/statements, “they are not restricted to 33 and can employ any number of items” (Brown, 2004, p. 4). The participants are asked to rank-order the Q statements into one single continuum under the statements *most unlike/neutral/ most like* respectively, according to their own perceptions and subjective thoughts based on instruction (Taylor et al., 1994, p. 173; Addams, 2000, p. 16; Stenner et al., 2017, p. 215). The Q statements are randomly numbered. The format used in the research is usually forced distribution or a “forced-choice research approach” (ten Klooster et al., 2008, p. 512) to capture participants’ perspective patterns. Forced distribution refers to the method by which participants are required to place each statement into the boxes to prevent any boxes from being left empty (Cross, 2005, p. 209) and that each position in the Q-sorting grid can be used only once (ten Klooster et al., 2008, p. 512). In addition, a range of values must be created for the Q-Sort index in the study, such as −3/+3, −4/+4, −5/+5, etc. The appropriate value range for the research is selected only after the pilot application of the research is conducted. In addition, as Brown (1980) indicates, the shape of the grid does not affect the examination of the results. While the Q statements that the participant deems most appropriate are placed in the + pole, the Q statements that the participant deems inappropriate are placed in the - pole (see Stephenson, 1980, p. 883; Brown, 1993, p. 102; Brown, 2004, p. 4; Ellingsen et al., 2010, pp. 399–400; Coogan and Herrington, 2011, pp. 25–26; Çırak Kurt and Yıldırım, 2018, p. 433; Trautmann et al., 2018, p. 395).

The research study is carried out according to the aforementioned essential principles of Q-methodology. First, given the fact that there is no specific rule or guideline concerning the number of statements or the range and distribution of shape of the Q-sorting grid (see Brown, 1971; Dennis, 1988; Mrtek et al., 1996; Addams, 2000; Amin, 2000; ten Klooster et al., 2008), 9 titles and 9 positive and 9 negative Q statements are arbitrarily formulated based on concourse following, i.e., Schlinger (1969). A pilot study is run on 7 people, and the value range of the Q-Sort index is determined to be between −3 and +3. This distribution is selected because, as Trautmann et al. (2018, p. 395) note, it “encourages participants to make fine discriminations between statements, particularly those that they may feel most strongly about.” At the following stage, the participants are asked to place Q statements in the Q-Sort index with the statements most unlike/neutral/most like. Forced distribution is used in the study; namely, the participants are informed not to leave any boxes empty.

The Q statements used in the study are formed based on assumptions of the socio-psychological theory of voter behaviour. While 9 of these statements, which we deem positive, are designed following the perspective of the socio-psychological approach, the remaining 9 statements, which we deem negative, include diametrically opposite statements and expressions against both the former 9 statements and the basic claims of the socio-psychological approach. Every title, therefore, consists of one positive and one negative statement and refers to certain socio-psychological factors, such as time factor, and political campaign and persuasion factors. In addition to this, the participants are also asked to write down whether they deem the structure and content of the Q statements inappropriate (Tables 2, 3).

**Table 2:** The Q-sorting grid with the “terms of reference” where participants are asked to rank the statements.

Most unlike			Neutral	Most like		
−3	−2	−1	0	1	2	3
						
						
		
										

**Table 3:** The Q titles and statements that participants are asked to rank on the Q-grid.

Titles	Statements
Time factor	I have a longstanding commitment to a certain political party. (9^1^) I support different political parties occasionally, for a long or short time. (13)
Central/local level factor	I vote for the same political party and/or its candidate in both central and local elections. (5) I vote for different political parties and/or candidates in central and local elections. (17)
Political participation factor	I make an effort to participate in the elections to vote for my preferred political party or candidate. (6) I do not mind participating in elections to vote for my preferred party. (14)
Political campaign and persuasion factor	Convincing political campaigns and policy proposals by political parties or the candidates I do not support encourage me to vote for them. (7) I do not care about the political campaigns and policy proposals of parties or the candidates I do not support aimed at convincing the voters. (10)
Candidate factor	Regardless of its/her little chance of winning the elections, I prefer to vote for the political party and/or the candidate I deem aligned with my beliefs and preferences. (2) Regardless of its/her image, I prefer to vote for the candidate with a higher chance of winning the elections. (18)
Rationality and irrationality factor	In elections, I prefer to vote for a political party and/or candidate according to my emotional affiliation or commitment. (8) In elections, I prefer to vote for a political party and/or candidate promising to implement the policies that convince me according to my own search and evaluation. (1)
Family factor	My family’s political views affect my decision to vote for a political party and/or candidate. (3) My family’s political views do not affect my decision to vote for a political party and/or candidate. (12)
Education factor	An increase in my education level has not affected my emotional connection with the political party and/or candidate I support. (15) As my education level has increased, my rational evaluation process has changed and this has affected my voting behaviour. (11)
Inner circle factor	If the majority of my inner circle support the same political party and/or the candidate, this affects my voting behaviour in favour of this party or candidate. (4) I vote in line with my own political views and evaluations only. (16)

1 Each number represents a statement which has been placed in the columns by the participants.

### 2.5 Data analysis

The data obtained from the Q-Sort index asked the participants to fill in are analysed using “PQMethod 2.35” (see Schmolck, 2014), the software programme prepared specifically for the Q methodology. All the data are manually transferred to the software programme, and then the factor analysis is conducted to identify the similarities between participants’ sorting of the statements in a holistic way. The main analysis is followed by performing a 10-degree hand rotation. After the hand rotation, participants clustering around common factors are marked with an X. Factor loadings illustrate each participant’s association with each of the identified socio-psychological factors and allow us to indicate (i) whether socio-psychological factors affect the voting behaviours of the participants who are politically educated and well-informed, (ii) if so, which socio-psychological factor(s) affects the voting behaviours of the participants. This also helps us to conclude that the participants who are mostly associated with one or more factors have similar voting behaviours corresponding to or in opposition to the assumptions of the socio-psychological approach.

After analysing the data, borrowing from Ayeb-Karlsson et al. (2020, p. 6), the statistical formula [=2.58 × (1 ÷ √*n*)] is used to determine the significance level. The significance value is calculated as 0.60, and a 10-degree hand rotation is performed.

## 3 Results

The number of factors identified as a result of the Principal Component Analysis (PCA) is 8. Even though no strict rules are available in Q methodology about how many factors ought to be extracted from the factor analysis (Coogan and Herrington, 2011, p. 26), the number of factors is reduced to 6 in this study as a result of a 10-degree varimax rotation. While 37 of the participants take a place under any factor, 20 do not. Participants are indicated by numbering p1, p2, …, p. 57 (Table 4).

**Table 4:** Factor loadings.

Participants/Factor loadings	1	2	3	4	5	6
p1	0.3746	0.5511	0.4100	0.0093	−0.1745	0.2508
p2	**0.9352X**	−0.0069	−0.1615	−0.0120	0.0340	0.0371
p3	−0.2815	**0.6294X**	−0.1833	0.1799	0.4876	−0.1651
p4	0.3566	0.5889	0.2965	0.2340	0.2471	−0.0307
p5	**0.8611X**	−0.0685	−0.1174	−0.0371	0.0404	−0.2691
p6	0.4656	−0.1783	−0.5270	0.0257	0.0815	0.3202
p7	**0.8045X**	0.3203	−0.2087	−0.0468	0.2591	0.1762
p8	**0.7827X**	−0.0084	−0.1951	0.3794	−0.1712	−0.2046
p9	−0.1723	0.5878	−0.1797	−0.3422	0.4244	0.0768
p10	**0.7117X**	0.1344	−0.3774	−0.0927	0.1152	−0.0466
p11	**0.8024X**	0.2477	−0.4232	−0.0001	0.0327	−0.0395
p12	−0.4786	**0.8093X**	−0.1752	−0.1035	−0.2013	−0.0578
p13	−0.4634	**0.7366X**	−0.1411	−0.3170	0.1058	0.0187
p14	0.5619	0.1788	−0.4434	0.4199	0.3294	−0.0317
p15	−0.1049	**0.6367X**	0.0836	0.2653	−0.3222	0.1290
p16	−0.3182	0.2878	0.0019	0.0871	**0.6399X**	0.2662
p17	0.5117	0.4289	0.5099	−0.2901	−0.0688	−0.2834
p18	0.4852	0.5781	0.3045	0.2432	0.2173	−0.0185
p19	−0.4786	**0.8093X**	−0.1752	−0.1035	−0.2013	−0.0578
p20	**0.7739X**	0.1932	−0.0945	0.0670	−0.2064	−0.3370
p21	0.1944	0.5735	0.4877	−0.1740	−0.3648	0.1903
p22	0.0906	0.4472	0.2066	**0.6454X**	0.1309	−0.2720
p23	−0.4634	**0.7366X**	−0.1411	−0.3170	0.1058	0.0187
p24	**−0.6348X**	0.5968	0.0277	0.3152	−0.1414	0.0892
p25	−0.1243	0.3810	−0.0138	**0.7557X**	0.3429	0.0702
p26	**0.6869X**	0.4202	−0.1544	−0.0871	−0.2381	−0.2096
p27	−0.1806	**0.7716X**	−0.0444	−0.0540	−0.2327	−0.1940
p28	0.4782	0.4841	−0.1970	0.1239	0.3766	0.3527
p29	**0.7451X**	0.0663	−0.1833	−0.4218	0.0749	−0.1146
p30	0.5189	0.2095	0.3087	0.1235	−0.1506	**0.6289X**
p31	−0.5582	0.5165	−0.0266	0.1582	−0.0897	−0.3062
p32	**0.7340X**	0.0156	−0.0622	−0.4390	−0.2741	−0.0285
p33	0.5692	**0.6437X**	0.3318	0.2562	0.0210	−0.0587
p34	**0.8539X**	−0.1657	0.0013	−0.1233	0.0311	−0.0190
p35	**0.8075X**	0.0225	−0.4563	0.0171	0.1187	−0.0679
p36	0.6040	0.0539	0.3920	0.2324	−0.0304	−0.0450
p37	−0.5908	0.6035	0.0113	−0.0667	0.0962	0.1772
p38	0.4353	**0.7340X**	0.1567	−0.1873	0.2530	0.0261
p39	**0.7796X**	0.2810	0.0748	0.1524	−0.2524	−0.0834
p40	−0.1830	0.5489	0.1665	0.2224	0.1886	−0.1428
p41	0.5432	0.0244	0.0606	−0.5795	0.4415	−0.1372
p42	**0.8449X**	0.0922	0.0159	−0.2763	0.0560	−0.2537
p43	0.4546	0.2025	−0.2149	0.0258	−0.2150	0.4917
p44	−0.4706	**0.7527X**	−0.2171	−0.1713	−0.2034	0.0322
p45	−0.4786	**0.8093X**	−0.1752	−0.1035	−0.2013	−0.0578
p46	−0.4786	**0.8093X**	−0.1752	−0.1035	−0.2013	−0.0578
p47	−0.5853	**0.7223X**	0.0415	−0.1396	0.1408	0.0046
p48	−0.4679	**0.7785X**	−0.2530	−0.1948	−0.1527	0.0026
p49	0.1602	0.1038	−0.3982	−0.1186	−0.1007	**0.6751X**
p50	**0.6333X**	−0.0736	0.0981	0.1480	−0.1547	0.1191
p51	0.5473	0.5519	0.3654	−0.0967	0.1714	−0.0852
p52	0.4565	0.4337	−0.0970	−0.1736	−0.0991	−0.1093
p53	0.5574	0.3878	0.2331	0.1049	−0.2253	0.3403
p54	0.1334	−0.1451	**0.7812X**	−0.3462	0.1586	0.0144
p55	0.1737	0.0117	0.3381	−0.1804	0.0811	0.4770
p56	**0.6833X**	−0.2151	−0.0824	0.0759	−0.2572	0.0638
p57	0.1301	0.1989	−0.3105	0.3607	−0.5115	−0.0167

A bold “X” indicates the similar perceptions of the participants loaded on certain factor loads.

Table 4 includes 57 participants belonging to the sample group of the study. A bold “X” indicates the similar perceptions of the participants loaded on certain factor loads. In other words, “X” shows which participant is loaded in which factor. It can be seen on the Table 4 that 17 people are loaded in Factor 1, 14 people in Factor 2, 1 person in Factor 3, 2 people in Factor 4, 1 person in Factor 5, and 2 people in Factor 6. The participants loaded in Factor 1 constitute 30% of the total group, and the participants loaded in Factor 2 constitute 25% of the total participants. For this reason, it can be stated that participants loaded in Factor 1 and Factor 2 form two separate groups and associate with two separate socio-psychological or otherwise factors on which they come into agreement. An evaluation is made on which common factor both groups have agreed on and which statements they attach much more importance to. Table 5 includes the statements, the Z value of the statements, and the Z-score and ranking of the statements for each factor. The statements are listed according to the order of Q statements given above in Table 3.

**Table 5:** Z-scores and order of importance of statements.

Factors Statements	Factor 1	Factor 2	Factor 3	Factor 4	Factor 5	Factor 6
	Z. Ranking	Z. Ranking	Z. Ranking	Z. Ranking	Z. Ranking	Z. Ranking
If the majority of my inner circle support the same political party and/or the candidate, this affects my voting behaviour in favour of this party or candidate.	−1.60 18	0.91 3	−1.62 17	1.11 3	1.08 3	−0.92 16
I vote in line with my own political views and evaluations only.	1.34 3	0.15 8	0.54 7	0.74 4	0.00 11	2.02 2
An increase in my education level has not affected my emotional connection with the political party and/or candidate I support.	−0.39 10	0.49 7	0.00 9	−1.82 18	1.62 2	0.11 8
As my education level has increased, my rational evaluation process has changed and this has affected my voting behaviour.	1.07 4	−0.49 13	0.00 8	1.82 1	0.54 7	0.67 4
My family’s political views have effects on my decision to vote for a political party and/or candidate.	−0.59 12	1.88 1	−1.08 16	1.44 2	1.62 1	0.61 5
My family’s political views do not affect my decision to vote for a political party and/or candidate.	1.35 2	−1.50 17	1.62 1	−1.11 16	0.00 9	−0.67 14
In elections, I prefer to vote for a political party and/or candidate according to my emotional affiliation or commitment.	−1.00 15	0.75 6	0.54 6	0.09 10	−1.62 17	−1.35 17
In elections, I prefer to vote for a political party and/or candidate promising to implement the policies that convince me according to my own search and evaluation.	1.64 1	0.10 9	−0.54 14	−1.21 17	−1.62 18	2.02 1
Regardless of its/her little chance of winning the elections, I prefer to vote for the political party and/or the candidate I deem aligned with my beliefs and preferences.	0.96 5	0.76 5	−0.54 13	0.60 6	0.00 10	−1.66 18
Regardless of its/her image, I prefer to vote for the candidate with a higher chance of winning the elections.	−0.43 11	−1.83 18	0.00 10	−0.70 13	−0.54 15	−0.79 15
I do not care about the political campaigns and policy proposals of parties or the candidates I do not support aimed at convincing the voters.	−1.17 16	−0.86 14	1.62 2	0.37 8	1.08 4	0.67 3
Convincing political campaigns and policy proposals by political parties or the candidates I do not support encourage me to vote for them.	0.31 8	−0.13 10	−1.62 18	−0.60 13	−0.54 13	0.36 7
I make an effort to participate in the elections to vote for my preferred political party or candidate.	0.26 9	0.82 4	1.08 3	0.60 6	0.54 6	−0.48 13
I do not mind participating in elections to vote for my preferred party.	−0.62 13	−0.37 12	−0.54 12	−0.23 11	−1.08 16	0.42 6
I have a longstanding commitment to a certain political party.	−1.19 17	1.44 2	1.08 4	−0.84 14	0.00 8	−0.00 9
I support different political parties occasionally, for a long or short time.	0.38 7	0.87 15	−1.08 15	0.46 7	−0.54 12	−0.36 11
I vote for the same political party and/or its candidate in both central and local elections.	−0.76 14	−0.33 11	0.54 5	0.23 9	0.54 5	−0.30 10
I vote for different political parties and/or candidates in central and local elections.	0.45 6	−0.89 16	0.00 11	−0.98 15	−1.08 14	−0.36 12

The rankings/order of the statements for all factors in the Table 5 indicate that the distinguishing statement, which is ranked most positively by the 17 participants located under Factor 1, is “[i]n elections, I prefer to vote for a political party and/or candidate promising to implement the policies that convince me according to my own search and evaluation.” The distinguishing statement which is ranked most negatively by the same group is “[i]f the majority of my inner circle support the same political party and/or the candidate, this affects my voting behaviour in favour of this party or candidate.” On the contrary, while the 14 participants located under Factor 2 rank the statement “[m]y family’s political views have effects on my decision to vote for a political party and/or candidate” most positively, they rank the statement “[r]egardless of its/her image, I prefer to vote for the candidate with a higher chance of winning the elections.”

The distinguishing statements ranked most positively by the participants clustering around Factor 1 in the second and third places are as follows, respectively: “My family’s political views have no effect on my decision to vote for a political party and/or candidate” and “I vote in line with my own political views and evaluations only.” On the contrary, the second and third most positively ranked statements by the participants clustering around Factor 2 are as follows, respectively: “I have a longstanding commitment to a certain political party” and “[i]f the majority of my inner circle support the same political party and/or the candidate, this affects my voting behaviour in favour of this party or candidate.” Whereas the participants loaded under Factor 1 rank “[i]f the majority of my inner circle support the same political party and/or the candidate, this affects my voting behaviour in favour of this party or candidate” as the most negative statement, “[r]egardless of its/her image, I prefer to vote for the candidate with a higher chance of winning the elections” is ranked as the most negative statement by the participants loaded under Factor 2.

It can be deduced that the first three statements that the participants rank most positively by the participants gathered under Factor 2 correspond to the assumptions of the socio-psychological approach. In other words, these participants’ voting behaviours are affected by some socio-psychological factors. However, given the first three statements ranked positively by the participants taking place under Factor 1, it can be assumed that they make their political decisions more rationally without being affected by socio-psychological factors and are ready to vote for alternative candidates and political parties. Suppose that if the participants under Factor 1 and Factor 2 are at opposite ends of the spectrum, whose voting behaviours are affected by either rational or socio-psychological factors, respectively, the voting behaviours of the participants gathered under no factors are affected by relatively “mixed” factors and take different places on the spectrum between the socio-psychological and rational approaches.

All the other statements that differentiate the opinions of the participants loaded under Factors 1 and 2 are given in the Table 6. As seen in the Table 6, the titles that differentiate the preferences of the participants cluster around both Factors 1 and 2 can be listed as *family, education, and rationality*. The Z-scores of the statements that are preferred differently between the two groups and that have a high significance value are marked in bold in the table. The statement on which the two groups disagree most strongly is “[m]y family’s political views do not affect my decision to vote for a political party and/or candidate.” Accordingly, it can be stated that the order and effect of priority of the socio-psychological factors shaping the voting behaviours of the participants loaded under Factors 1 and 2 are different. The order of priority of factors affecting the voting behaviour of the participants under Factor 1 is individual search and evaluations, promises/actions, a short-term commitment to, and the image of the political party and/or the candidate. The order of priority of factors affecting the voting behaviour of the participants under Factor 2, on the contrary, is loyalty to her family’s and inner circles’ political preferences, and a long-term commitment, and an emotional attachment to a political party and/or the candidate. On the other hand, the titles on which the two groups agree most are *candidate and education*. In both factors, “[r]egardless of its/her little chance of winning the elections, I prefer to vote for the political party and/or the candidate I deem aligned with my beliefs and preferences” is the fifth most positively ranked statement. Given the title of education, a differentiation between the groups is observed despite the aforementioned agreement. While the participants loaded under Factor 1 deem the statement “[a]s my education level has increased, my rational evaluation process has changed, and this has affected my voting behaviour” more positively, the participants loaded under Factor 2 regard “[i]ncrease in my education level has not affected my emotional connection with the political party and/or candidate I support” as a much more positive statement.

**Table 6:** Z-scores of the statement rankings of the participants loaded under Factor 1 and Factor 2.

Statements	Z-Score
My family’s political views do not affect my decision to vote for a political party and/or candidate.	**2.86**
As my education level has increased, my rational evaluation process has changed and this has affected my voting behaviour.	**1.56**
In elections, I prefer to vote for a political party and/or candidate promising to implement the policies that convince me according to my own search and evaluation.	**1.53**
Regardless of its/her image, I prefer to vote for the candidate with a higher chance of winning the elections.	**1.39**
I vote for different political parties and/or candidates in central and local elections.	**1.35**
I support different political parties occasionally, for a long or short time.	**1.26**
I vote in line with my own political views and evaluations only.	**1.19**
I do not care about the political campaigns and policy proposals of parties or the candidates I do not support aimed at convincing the voters.	0.44
Regardless of its/her little chance of winning the elections, I prefer to vote for the political party and/or the candidate I deem aligned with my beliefs and preferences.	0.19
I do not mind participating in elections to vote for my preferred party.	−0.25
Convincing political campaigns and policy proposals by political parties or the candidates I do not support encourage me to vote for them.	−0.31
I vote for the same political party and/or its candidate in both central and local elections.	−0.43
I make an effort to participate in the elections to vote for my preferred political party or candidate.	−0.55
An increase in my education level has not affected my emotional connection with the political party and/or candidate I support.	−0.88
In elections, I prefer to vote for a political party and/or candidate according to my emotional affiliation or commitment.	**−1.75**
My family’s political views have effects on my decision to vote for a political party and/or candidate.	**−2.47**
If the majority of my inner circle support the same political party and/or the candidate, this affects my voting behaviour in favour of this party or candidate.	**−2.51**
I have a longstanding commitment to a certain political party.	**−2.63**

To reveal the titles that are given the highest priority according to the factors, for each factor, the Z-score is averaged, covering all the titles. Borrowing from Yıldırım (2017), the following formula is used for the calculation: Z-average = (Z-score of the positive statement related to the title – Z-score of the negative statement related to the title)/2 (Yıldırım, 2017). See Table 7.

**Table 7:** The overall Z-score weighted average of the titles of the Q statements.

Titles of the Q Statements	Factor 1 17 Participants	Factor 2 14 Participants	Factor 3 1 Participants	Factor 4 2 Participants	Factor 5 1 Participants	Factor 6 2 Participants	Weighted average
*Time Factor*	−0.78	0.28	1.08	0.65	0.27	0.18	−0.17
*Central/Local Level Factor*	−0.60	0.28	0.27	0.60	0.81	0.03	−0.10
*Political Participation Factor*	0.44	0.59	0.81	0.41	0.81	0.45	0.52
*Political Campaign and Persuasion Factor*	−0.74	−0.36	1.62	0.48	0.81	0.15	−0.38
*The Candidate Factor*	−0.69	1.29	−0.27	0.65	0.27	−0.43	0.18
*Rationality & Irrationality Factor*	−1.32	0.32	0.54	0.65	0.00	−1.68	−0.53
*Family Factor*	−0.97	1.69	−1.35	1.27	0.81	0.64	0.28
*Education Factor*	−0.73	0.49	0.00	−1.82	0.64	−0.56	−0.26
*Inner Circle Factor*	−1.47	0.38	−1.08	0.18	0.54	−1.47	−0.62

The titles on which 37 participants, who are loaded under all factors, agree most and which are the most positively ranked are the political participation factor (Xz = 0.52), family factor (Xz = 0.28), and candidate factor (Xz = 0.18). The titles on which participants disagree most and which are the most negatively ranked are the inner circle factor (Xz = −0.62), rationality and irrationality factor (Xz = −0.53), and political campaign and persuasion factor (Xz = −0.32).

## 4 Limitations

Two main limitations of this study can be noted: first is the size of the sample group. Even though the Q-methodology allows researchers to do an empirical study with a small sample of participants (Dennis, 1988), the sample is selected from only one university’s postgraduate students of Political Science and Public Administration, and all the postgraduate students of this department could not participate in the research due to some technical difficulties. 62 out of 82 postgraduate students accepted to participate in the Q methodology study; 57 of them completed and handed in the Q-grid; and 5 students did not complete the study. Accordingly, the percentage of subjects who agreed to participate is 75.6%, and the percentage who completed the survey is 91.93%. Second, other theories of voter behaviour, such as rational voter behaviour, sociological theory of voter behaviour, etc., are excluded within the scope of the study — as seen above, these theories are mentioned when needed.

Other limitations are mostly related to some additional/complementary methodological tools that are not used in the study but which, if used, would have improved the quality of the study and the validity of its results: vignettes, for instance, are not used in this study. In some field studies, vignettes are used to create a well-designed experimental environment in order to accurately frame and convey different future possibilities to participants. The works of Birch and Allen (2015), Gerber et al. (2016), Carreras and Vera (2018), Brown et al. (2019), Schafheitle et al. (2020), Hurwitz et al. (2022), Noordzij et al. (2023), and Zhou (2023) are some examples of the use of vignettes. In this study, however, the participants are not provided with different possible future scenarios in the judgement sentences.

Moreover, in modern democracies, the economy is one of the most important factors that play a role in the formation of voter preferences. Economic factors affecting voting behaviour can be on a micro scale, such as the financial wealth of the individual, or on a macro scale, such as the general economic situation of the country. The scholarly literature is replete with studies revealing either that a voter’s personal financial situation affects voting behaviour (see Markus, 1988; Brooks and Brady, 1999; Leigh, 2005; Lind, 2007) or that national economic performance, such as unemployment, inflation, and growth, affect voting behaviour (see Powell and Whitten, 1993; Anderson, 2000; Lewis-Beck and Stegmaier, 2000; Lewis-Beck and Nadeau, 2011; Fossati, 2014; Hansford and Gomez, 2015; Ataay, 2020). In this study, which focuses specifically on the socio-psychological factors affecting voting behaviour, data on participants’ financial wealth are not requested from the participants. Since Q methodology is applied to postgraduate students who are assumed to have no or average personal income (ignoring the financial resources provided to students by their families or other individuals or institutions), it is assumed that the aforementioned effect does not function in our case. Accordingly, the impact of the financial status of individual and national economic indicators on voting behaviour is ignored.

For a similar reason, incentivised experimental tools (see Orcutt and Orcutt, 1968; Smith and Walker, 1993; Camerer and Hogarth, 1999; Hertwig and Ortmann, 2001; Gaechter and Renner, 2010; Dressler and Mugerman, 2023), which are mostly used to identify economic factors that may affect voting behaviour, are not also used in this study. In other words, this study is conducted through the Q methodology within the framework of non-incentivised experimental approaches (for an illustrative example of a study that employs a non-incentivised approach, see Charness et al., 2021). This is because this study aims to assess postgraduate students’ self-attitudes towards voting behaviour in a way that is immune from material factors that may have a direct impact on them.

Finally, even though, for instance, Libby and Rennekamp’s (2012), Kachelmeier et al.’s (2020), and Mugerman et al.’s (2020) works exemplify and highlight the importance of “soft approaches,” like a combination of experiments and surveys, in studying individual decision-making, an experiment is not conducted in our study. Future studies on the factors affecting voting behaviour can combine the Q methodology with the experiment. In this way, a new method can be tested, and new conclusions and inferences can be drawn from the findings obtained with this new method.

## 5 Conclusion

This research project aims to identify similar or different perspectives of postgraduate students at Akdeniz University, Department of Political Science and Public Administration, on the socio-psychological factors affecting their voting behaviour. For this purpose, a Q-sort study is utilised to discover the attitudinal groupings and clusters of respondents, including confounding and non-significant respondents.

This analysis concludes that 37 participants are gathered under 6 different factors, but 20 do not load on any factor. Among those 6 factors, the factor groups with the highest number of participants are Factor 1 with 17 participants and Factor 2 with 14 participants. It can be observed that the number of participants with mixed views is higher than the number loaded on Factors 1 and 2 separately.

While participants who load on Factor 1 agree on statements about making political decisions rationally and being ready to vote for alternative candidates and political parties in different elections, those who load on Factor 2 have consensus on statements about making political decisions affected by some socio-psychological factors. The participants gathered under no factors, however, seem to be affected by relatively mixed factors while making their political decisions.

The titles that 37 participants loaded on all factors approach most positively are *political participation, family, and the candidate*. In other words, this result shows that while placing the Q statements in the index, the participants give the highest importance to these three titles. Participants loaded on Factor 1, who acted more rationally in their political preferences, differ only in the title of *family*. The title of the *candidate* epitomises a socio-psychological factor that the participants clustering around both Factors 1 and 2 are highly associated with. On the other hand, the titles that differentiate the preferences of the participants cluster around both Factors 1 and 2 can be listed as *family, education, and rationality.*

In addition, a relationship is found between the education factor and the emotional voter. This finding is an expected result within the scope of the socio-psychological approach (see Campbell et al., 1960). To explain, the participants loaded on Factor 1, who exhibit a rational approach, agree on the statement that as their education level increases, their voting behaviour is more affected by rational factors. This result supports Gülmen’s (1979) claim that an increase in the education level of the electorate rationalises the electorate’s voting behaviour. On the other hand, the participants loaded on Factor 2, who exhibit a less rational approach (in other words, they are not totally less/irrational or unwilling to vote for alternative candidates; they just cluster around Factor 2 and are inclined to behave in an emotional way), agree with the view that an increase in their education level does not affect their emotional ties to the political party or candidate that they have been supporting. Given the aforementioned relationship between the education factor and the emotional voter, *political participation* is also revealed as the most positively approached title of all factors. This result complies with Burden’s (2009, p. 542) argument that an increase in education level increases the rate of political participation. Similarly, regardless of the rational or emotional profile of the voter, it can be stated that another title taken into consideration by all participants is the candidate’s image factor.

In sum, this study concludes that the majority of the participants constitute three groups of voters who act rationally, emotionally, or both rationally and emotionally while making their political decisions. In other words, the voting behaviours of the majority of postgraduate students, a voter group who are politically educated and well-informed about voting behaviours, are affected by both rational and socio-psychological factors. It should, however, be noted that rationality and emotion are not two independent cognitive faculties. This distinction is made by individuals in a phenomenological or hermeneutic way. The results of this study can also be interpreted by some in this way. The reason why there is no information or assumption about why and when those psychological factors come into effect can be one of the weaknesses of the Q-sort methodology.

Despite the criticism mentioned above, the Q-sort methodology is a suitable tool for exploring both rational and socio-psychological factors affecting voting behaviours. It is expected that the results obtained with the Q-study in this research project might provide a ground for further studies aiming to detect similar or different factors, tendencies, and perceptions of the voter groups.

## Data availability statement

The original contributions presented in the study are included in the article/supplementary material, further inquiries can be directed to the corresponding author.

## Ethics statement

The studies involving human participants were reviewed and approved by the Akdeniz University ethics committee. The participants provided their written informed consent to participate in this study.

## Author contributions

TŞ contributed to the conception of the study and to developing the research design. YB helped develop the research design, performed all different stages of the Q methodology analysis, and contributed to writing the manuscript. YA contributed to the conception of the study and to writing the manuscript. SD helped apply the method to the practical example. KO and SZ helped write the manuscript. All authors contributed to the article and approved the submitted version.
